# Information for Persons Who Are Immunocompromised Regarding Prevention and Treatment of SARS-CoV-2 Infection in the Context of Currently Circulating Omicron Sublineages — United States, January 2023

**DOI:** 10.15585/mmwr.mm7205e3

**Published:** 2023-02-03

**Authors:** Pragna Patel, Evelyn Twentyman, Emily Koumans, Hannah Rosenblum, Shannon Griffin-Blake, Brendan Jackson, Sara Vagi

**Affiliations:** 1 CDC COVID-19 Emergency Response Team.

As of January 20, 2023, >90% of circulating SARS-CoV-2 variants in the United States, specifically Omicron BQ.1, BQ.1.1, XBB, and XBB.1.5 sublineages, are unlikely to be susceptible to the combined monoclonal antibodies, tixagevimab and cilgavimab (Evusheld) used for preexposure prophylaxis against SARS-CoV-2 infection (*1*). The Food and Drug Administration announced on January 26, 2023, that Evusheld is not currently authorized for preexposure prophylaxis against SARS-CoV-2 infection in the United States (*2*). It is important that persons who are moderately to severely immunocompromised,* those who might have an inadequate immune response to COVID-19 vaccination, and those with contraindications to receipt of COVID-19 vaccines, exercise caution and recognize the need for additional preventive measures (Box). In addition, persons should have a care plan that includes prompt testing at the onset of COVID-19 symptoms and rapid access to antivirals if SARS-CoV-2 infection is detected.

BOX **Prevention measures against SARS-CoV-2 for persons who are immunocompromised, their household members, and close contacts in the context of currently circulating Omicron sublineages — United States, January 2023**

Because Evusheld is not currently authorized for preexposure prophylaxis against SARS-CoV-2 infection in the United States, it is important that persons who are moderately to severely immunocompromised,* those who might have an inadequate immune response to COVID-19 vaccination, and those with contraindications to receipt of COVID-19 vaccines, exercise caution and recognize the need for additional preventive measures to protect themselves from SARS-CoV-2 infection. Persons with immunocompromise, their household members, and close contacts can use the following steps and precautions to help prevent SARS-CoV-2 infection and mitigate COVID-19 illness if they become infected.

COVID-19 vaccines, booster doses, and staying up to date*

- COVID-19 vaccines remain the best way to protect against severe COVID-19. COVID-19 vaccines help the body develop protection against SARS-CoV-2 infection. Although vaccinated persons sometimes get infected with SARS-CoV-2, staying up to date with COVID-19 vaccines significantly lowers the risk for severe illness, hospitalization, or death from COVID-19.
- CDC recommends that all persons who are eligible, especially those who are immunocompromised or have weakened immune systems,^†^ get an updated (bivalent) booster dose and stay up to date with their COVID-19 vaccines.

**Personal COVID-19 action plan^§^**

- Persons should consider how to protect themselves and others around them should they become ill with COVID-19 or if the community COVID-19 transmission level changes. The plan should include:
  - ways to protect oneself and others including considerations in case of illness, such as finding a room in which to isolate
  - actions to take in case of exposure or symptom onset
  - what to do in the event of receipt of a positive SARS-CoV-2 test result
- Persons should share their COVID-19 plan with their family, friends, and health care providers so they can support prevention and preparation steps. CDC suggests that persons consider how others can help them if they get ill. It is important to adhere to treatment plans, keep routine health care appointments, and ensure that prescriptions are filled. Persons should make alternative plans for work, child care, and other responsibilities that might cause stress if they become ill.

**Masks or respirators^¶^**

- Masks are made to contain droplets and particles that persons breathe, cough, or sneeze. A variety of masks are available. Some masks provide a higher level of protection than others. Wearing a mask with the best fit and comfort provides the best protection.**
- Respirators (e.g., N95 and NIOSH-approved KN95) provide higher protection than masks.^††^ Respirators are made to protect persons by fitting closely on their face to filter out particles, including SARS-CoV-2. They can also block droplets and particles that a person breathes, coughs, or sneezes out to limit transmission to others. NIOSH approves many types of filtering facepiece respirators. The most widely available are N95 respirators, but other types (N99, N100, P95, P99, P100, R95, R99, and R100) offer the same or better protection as an N95 respirator.

**Physical distancing**

Small particles that persons breathe out can contain virus particles. The closer a person is to other persons, the higher the risk for exposure to SARS-CoV-2. Persons can minimize risk of exposure by avoiding indoor crowded areas or maintaining a ≥6 ft (1.8 m) distance from others. Such actions must be balanced against risks of avoiding such activities.

**Ventilation^§§^**

- Opening windows and doors to bring as much fresh air into the home as possible (weather permitting) can improve ventilation.
- Portable high-efficiency particulate air cleaners are useful if a home is not outfitted with an HVAC system.
- Exhaust fans and other fans can improve air flow.
- In homes where the HVAC fan operation can be controlled by a thermostat, the fan should be set to the “on” position instead of “auto” when others are visiting. This allows the fan to run continuously, even if heating or air conditioning is not on, to ensure the HVAC system provides continuous airflow and filtration.

**Time outdoors**

Spending time outdoors, when possible, instead of indoors, can also help reduce transmission. Viral particles spread between persons more readily indoors than outdoors.

**Handwashing**

Frequent handwashing with soap and water, preferably, or using a hand sanitizer that contains ≥60% alcohol can reduce risk for many illnesses, including COVID-19.

**Testing for SARS-CoV-2^¶¶^**

- Persons should get tested if they have COVID-19 symptoms. Viral tests are used for SARS-CoV-2 detection. There are two types of viral tests: rapid tests and laboratory tests. These tests might use nasal, throat, or saliva samples. Persons can take actions to reduce further transmission if they are aware of their SARS-CoV-2 infection.
- Free at-home tests*** are available. Persons should check with their health insurance, Medicaid, or Medicare plan to learn what tests are available.^†††^ Persons with a disability can receive help from the Disability Information and Access Line^§§§^ to access a test or identify an accessible test location.
- Persons should be aware of free or low-cost testing locations^¶¶¶^ that are near their homes.

COVID-19 Treatment****

- Persons should contact their health care provider, health department, or community health center^††††^ to learn about treatment options. Treatment must be started within 5–7 days after symptoms develop to be effective.
- Community Test to Treat locations^§§§§^ can be accessed if or when persons cannot reach their health care provider or do not have one. These sites offer testing and prescriptions from a health care provider (either onsite or by telehealth) and dispense medications.
- Antiviral treatments are available for persons with mild to moderate COVID-19 symptoms who are at high risk for progression to severe disease, hospitalization, and death. Persons are at high risk of disease if they
  - are aged ≥50 years
  - have an underlying health condition,^¶¶¶¶^ especially moderate to severe immunosuppression
  - are unvaccinated
- Persons who are immunocompromised should discuss a treatment plan with their doctor and identify which COVID-19 treatment would be best for them. Some persons with COVID-19 who are immunocompromised or receiving immunosuppressive treatment might benefit from a convalescent plasma treatment.*****
- CDC recommends that immunocompromised persons with COVID-19 isolate for ≥10 days and check with their health care provider before ending isolation.^†††††^

**Abbreviations:** HVAC = heating, ventilation, and air conditioning; NIOSH = National Institute for Occupational Safety and Health.

* https://www.cdc.gov/coronavirus/2019-ncov/vaccines/stay-up-to-date.html

^†^ https://www.cdc.gov/coronavirus/2019-ncov/need-extra-precautions/people-with-medical-conditions.html

^§^ https://www.cdc.gov/coronavirus/2019-ncov/downloads/needs-extra-precautions/FS_COVID_Plan_FINAL.pdf

^¶^ https://www.cdc.gov/coronavirus/2019-ncov/prevent-getting-sick/types-of-masks.html

** Persons who are deaf or hard of hearing may request a clear mask to assist with lipreading or seeing facial expressions. Persons with sensory disorders or intellectual and developmental disabilities might be unable to wear masks and should consider face shields.

^††^ Persons with severe respiratory impairment (e.g., shortness of breath with minimal exertion or supplemental oxygen use) should consult with a health care provider regarding N95 respirator usage. Some N95 respirators might contain latex. Persons with natural rubber latex allergies should consult the manufacturer’s website for information about the specific model.

^§§^ https://www.cdc.gov/coronavirus/2019-ncov/prevent-getting-sick/Improving-Ventilation-Home.html; https://www.cdc.gov/coronavirus/2019-ncov/community/ventilation.html

^¶¶^ https://www.cdc.gov/coronavirus/2019-ncov/symptoms-testing/testing.html

*** https://special.usps.com/testkits

^†††^ https://www.cms.gov/how-to-get-your-at-home-OTC-COVID-19-test-for-free

^§§§^ https://acl.gov/DIAL

^¶¶¶^ https://www.hhs.gov/coronavirus/community-based-testing-sites/index.html

**** https://www.cdc.gov/coronavirus/2019-ncov/your-health/treatments-for-severe-illness.html

^††††^ https://data.hrsa.gov/data/reports/datagrid?gridName=FQHCs

^§§§§^ https://covid-19-test-to-treat-locator-dhhs.hub.arcgis.com/

^¶¶¶¶^ https://www.cdc.gov/coronavirus/2019-ncov/hcp/clinical-care/underlyingconditions.html

***** https://www.fda.gov/media/136798/download

^†††††^ https://www.cdc.gov/coronavirus/2019-ncov/your-health/isolation.html

COVID-19 vaccination remains the most effective way to prevent SARS-CoV-2–associated serious illness, hospitalization, and death. All persons, including those who are immunocompromised and their household members and close contacts, should stay up to date with COVID-19 vaccination, and receive the updated (bivalent) booster dose, when eligible.^†^ Although persons who are moderately to severely immunocompromised might not mount a strong vaccine-mediated immune response, staying up to date with COVID-19 vaccination^§^ does provide some protection (*3*,*4*). A recent CDC study of preliminary data showed that a bivalent booster dose provided additional protection against symptomatic SARS-CoV-2 infection among immunocompetent persons who had previously received 2, 3, or 4 monovalent vaccine doses (*4*).

Despite evidence of vaccine effectiveness, coverage with the bivalent booster dose across the United States remains low. As of January 18, 2023, 15.3% of persons aged ≥5 years had received a bivalent booster dose (*5*). CDC recommends that all eligible persons aged ≥6 months receive 1 bivalent booster dose. Persons are eligible for a bivalent booster dose if they are aged 6 months–5 years and have completed a Moderna COVID-19 primary series ≥2 months earlier. Persons aged 6 months–4 years and who received a 2-dose Pfizer COVID-19 primary series ≥8 weeks earlier can receive the bivalent booster as their third dose.

Among persons with immunocompromise and their household members and close contacts, prevention measures^¶^ including wearing a high-quality and well-fitting mask,** maintaining physical distance from others (≥6 ft [1.8 m]), improving indoor ventilation,^††^ practicing frequent handwashing, and developing a care plan,^§§^ should be considered in addition to receipt of a bivalent booster dose. It is important to wear a mask and maintain physical distance from others if it is not possible to avoid crowded indoor spaces. In addition, simple interventions should be used to improve ventilation in buildings and decrease SARS-CoV-2 transmission by improving air flow. CDC has developed interactive tools^¶¶^ to help identify ways to improve ventilation in the home. In-duct ultraviolet germicidal irradiation lights can also be added to home heating ventilation and air conditioning systems to inactivate SARS-CoV-2 as air passes through the system.*** Frequent handwashing with soap and water is the best way to eliminate germs in most situations. If soap and water are not readily available, an alcohol-based hand sanitizer containing ≥60% alcohol is a good alternative. Also, it is important for persons who are immunocompromised to develop a care plan in consultation with their physician, in the event that they develop COVID-19. 

Persons with mild to moderate symptoms of COVID-19 who 1) are aged ≥50 years, 2) have an underlying health condition^†††^ (especially moderate to severe immunosuppression), or 3) are unvaccinated are at risk for severe COVID-19–associated outcomes. Irrespective of vaccination status, symptomatic persons who are immunocompromised, their household members, and their close contacts should be tested for SARS-CoV-2 infection as soon as possible and receive treatment within 5–7 days of symptom onset. Early outpatient treatment of mild to moderate COVID-19 with a recommended first-line therapy, ritonavir-boosted nirmatrelvir (Paxlovid) or remdesivir (Veklury), or the second-line therapy, molnupiravir (Lagevrio), have been shown to reduce the risk for severe COVID-19, including hospitalization and death.^§§§^ These medications are expected to retain activity against the currently circulating Omicron sublineages (*6*) and are widely available.^¶¶¶^ Available COVID-19 treatment does not supplant the need for persons to stay up to date on their COVID-19 vaccinations, which are highly effective at preventing COVID-19–related morbidity and mortality.

## Footnotes

*
https://www.cdc.gov/coronavirus/2019-ncov/need-extra-precautions/people-who-are-immunocompromised.html#

†
https://www.cdc.gov/coronavirus/2019-ncov/vaccines/stay-up-to-date.html

§
https://www.cdc.gov/coronavirus/2019-ncov/vaccines/recommendations/immuno.html

¶
https://www.cdc.gov/coronavirus/2019-ncov/prevent-getting-sick/prevention.html

**
https://www.cdc.gov/coronavirus/2019-ncov/prevent-getting-sick/types-of-masks.html

††
https://www.cdc.gov/coronavirus/2019-ncov/prevent-getting-sick/improving-ventilation-home.html

§§
https://www.cdc.gov/coronavirus/2019-ncov/downloads/332440-A_FS_COVID_Plan_FINAL.pdf

¶¶
https://www.cdc.gov/coronavirus/2019-ncov/prevent-getting-sick/interactive-ventilation-tool.html

***
https://www.cdc.gov/coronavirus/2019-ncov/community/ventilation.html

†††
https://www.cdc.gov/coronavirus/2019-ncov/hcp/clinical-care/underlyingconditions.html

§§§
https://www.covid19treatmentguidelines.nih.gov/management/clinical-management-of-adults/

¶¶¶
https://aspr.hhs.gov/TestToTreat/Pages/default.aspx
